# Depersonalization and derealization as sequelae of a temporal lobe lesion: a case report

**DOI:** 10.1186/s12888-024-05641-2

**Published:** 2024-03-06

**Authors:** Jasmyn E. A. Cunningham, Caitlin S. R. Lees

**Affiliations:** 1 Department of Psychiatry, Faculty of Medicine, Dalhousie University, Halifax, NS Canada; 2 Division of Palliative Medicine, Department of Medicine, Faculty of Medicine, Dalhousie University, Halifax, NS Canada

**Keywords:** Case report, Depersonalization, Derealization, Brain metastasis, Temporal lobe, Palliative care, Dissociation, Brain tumor, Lung cancer

## Abstract

**Background:**

Depersonalization and derealization can occur not just from psychiatric causes but also from various organic etiologies, such as seizures and intracerebral structural abnormalities. However, there have been no previous reported cases to the authors’ knowledge detailing isolated depersonalization and derealization in the absence of clinical seizure activity or other psychiatric pathology, as sequelae of structural intracerebral lesions.

**Case presentation:**

In this case report, we detail the unique presentation of a 68-year-old woman under the care of palliative medicine who experienced depersonalization and derealization secondary to a metastatic lesion in her temporal lobe, in the parahippocampal gyrus to medial occipitotemporal gyrus region. These symptoms were present in the absence of any clinical seizure activity or other psychiatric symptomatology and largely resolved with the use of steroidal therapy, before returning secondary to disease progression.

**Conclusions:**

We discuss the relationship among isolated depersonalization and derealization with pathology of the left posterior temporal lobe in the context of this interesting case. This case expands our knowledge of the neurobiology of these phenomena, given the specific localization of the intracerebral pathology and temporal specificity of symptoms relative to tumor growth and treatment course.

## Background

Dissociation has been described in the psychiatric literature dating back to the mid- to late- 1800s [1], and is generally understood as a decreased ability to integrate information which would normally be connected, and/or the ability to integrate this information into consciousness [2]. According to the DSM-5, dissociation is a “disruption of and/or discontinuity in the normal integration of consciousness, memory, identity, emotion, perception, body representation, motor control, and behavior” [3]. Depersonalization/derealization disorder, one of the five DSM-5 dissociative disorders [3] also included in ICD-11 [4], involves persistent or recurrent episodes of either or both phenomena (but importantly, cannot be secondary to another medical condition or physiologic effects of a substance). Depersonalization, first coined by Dugas in 1898 as “*the state in which the self feels that its acts are strange and beyond its control*,” where “*mental states can become detached from their feeling of belonging to the self”* [5] is described in DSM-5 as “experiences of unreality, detachment, or being an outside observer with respect to one’s thoughts, feelings, sensations, body, or actions” (or “… with respect to surroundings” in the case of derealization) [3]. These experiences are distinct from psychosis, as an individual’s ability to discern reality stays fully intact; in addition to dissociative disorders, they are often seen in psychiatric syndromes such as post-traumatic stress disorder and borderline personality disorder, often relating to previous experiences of trauma [3]. Dissociation exists on a spectrum, from potentially serving adaptive functions to causing impairment in functioning and representing pathology (and thus comprising a dissociative disorder) [2, 3].

In addition to comprising part of various psychiatric syndromes, dissociation can also be seen as a result of various ‘organic’ states, including substance use, medication side-effects, and other medical conditions affecting neurologic functioning [2, 6–8]. Dissociation was first linked to temporal lobe epilepsy in the late 1800s, with subsequent data also showing temporal lobe encephalography (EEG) and other neurophysiological abnormalities in patients with dissociation and a history of trauma or other psychiatric comorbidities, and/or primary depersonalization disorder [2, 9, 10]. There have been reported cases of depersonalization and/or derealization in epilepsy, mostly associated with temporal lobe etiology [6–8, 11–13], with temporal lobe epilepsy having been proposed as a neurological model for the study of these phenomena given the similarly between ictal semiology and psychiatric presentation [8]. Derealization was described in the 1950s by Wilder Penfield et al. as a result of direct temporal lobe and limbic stimulation in patients with a history of epilepsy (and notably, was not produced by stimulation of other areas of the cortex), described as “illusions of recognition*”* [14, 15]; other groups have noted similar results [16, 17]. Depersonalization and derealization have also been reported in some case reports following traumatic brain injury [7, 18], migraine [7], and with other intracerebral pathologies including tumors [7, 15, 19–22]. However, in all of these previous cases, the neuroanatomical correlates of the dissociation are not clear, as the organic etiology is either not specifically localizable (e.g. epilepsy, migraine, traumatic brain injury) or tumors and cerebrovascular disease cases were complicated by concomitant epilepsy and/or other psychiatric symptoms [7, 15, 19–21].

We report here a unique case of a 68-year-old female patient who experienced depersonalization and derealization secondary to a metastatic lesion in her temporal lobe, in the parahippocampal gyrus to medial occipitotemporal gyrus region, in the absence of any clinical seizure activity or other psychiatric pathology.

## Case presentation

### Clinical course of cancer and dissociative experiences

**Fig. 1:**
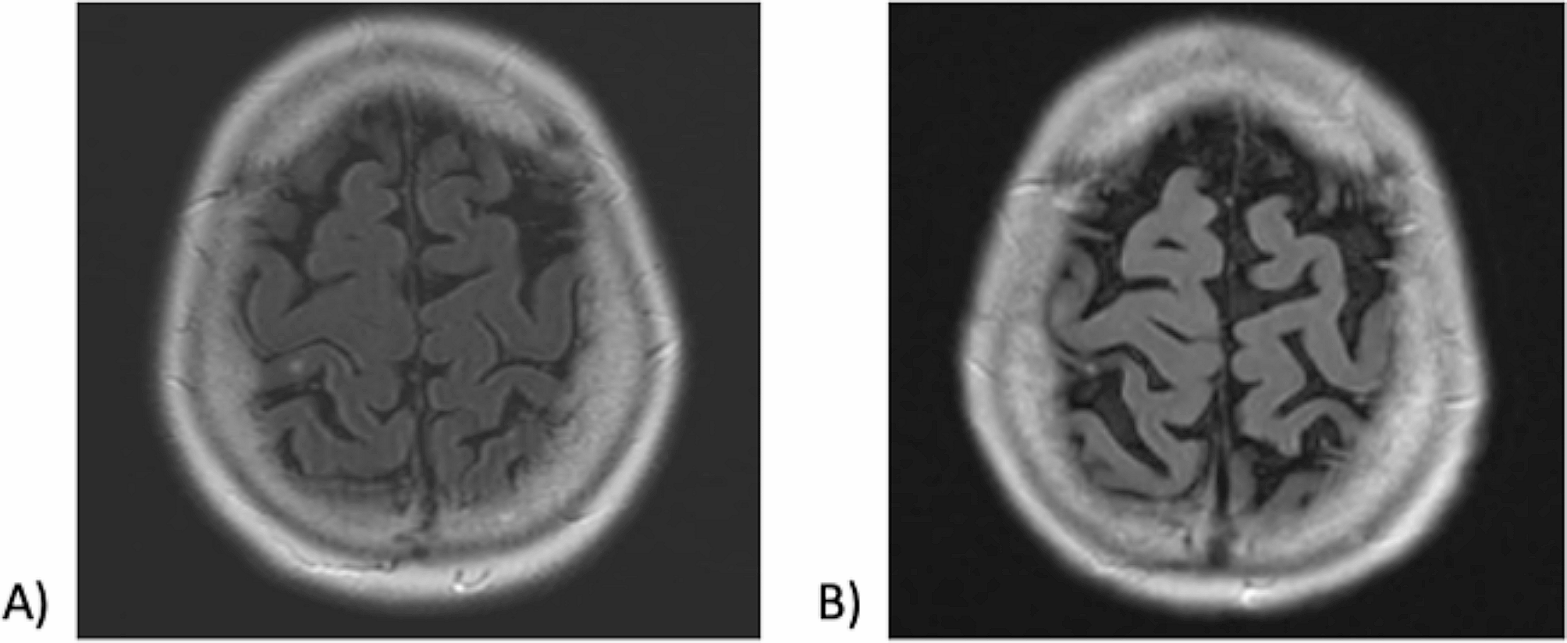
MRI Brain. Illustrates the stable right postcentral gyrus lesion, present on imaging since April 2021. (**A**) Axial T2 FLAIR with contrast, April 2021. (**B**) Axial T2 FLAIR post-gadolinium, Nov. 2022

The patient was a 68-year-old woman with lung cancer^1^ originally diagnosed in 2008, with recurrence four years later and again in 2021, even following several interventions including lobectomy, resections, chemotherapy, and radiation. Unfortunately, the patient then went on to develop metastatic disease including the brain– routine imaging for staging at this time showed a 2 mm right postcentral gyrus lesion (Fig. 1). She underwent further treatments including 3 cycles of first line systemic therapy and 11 cycles of maintenance therapy.

**Fig. 2:**
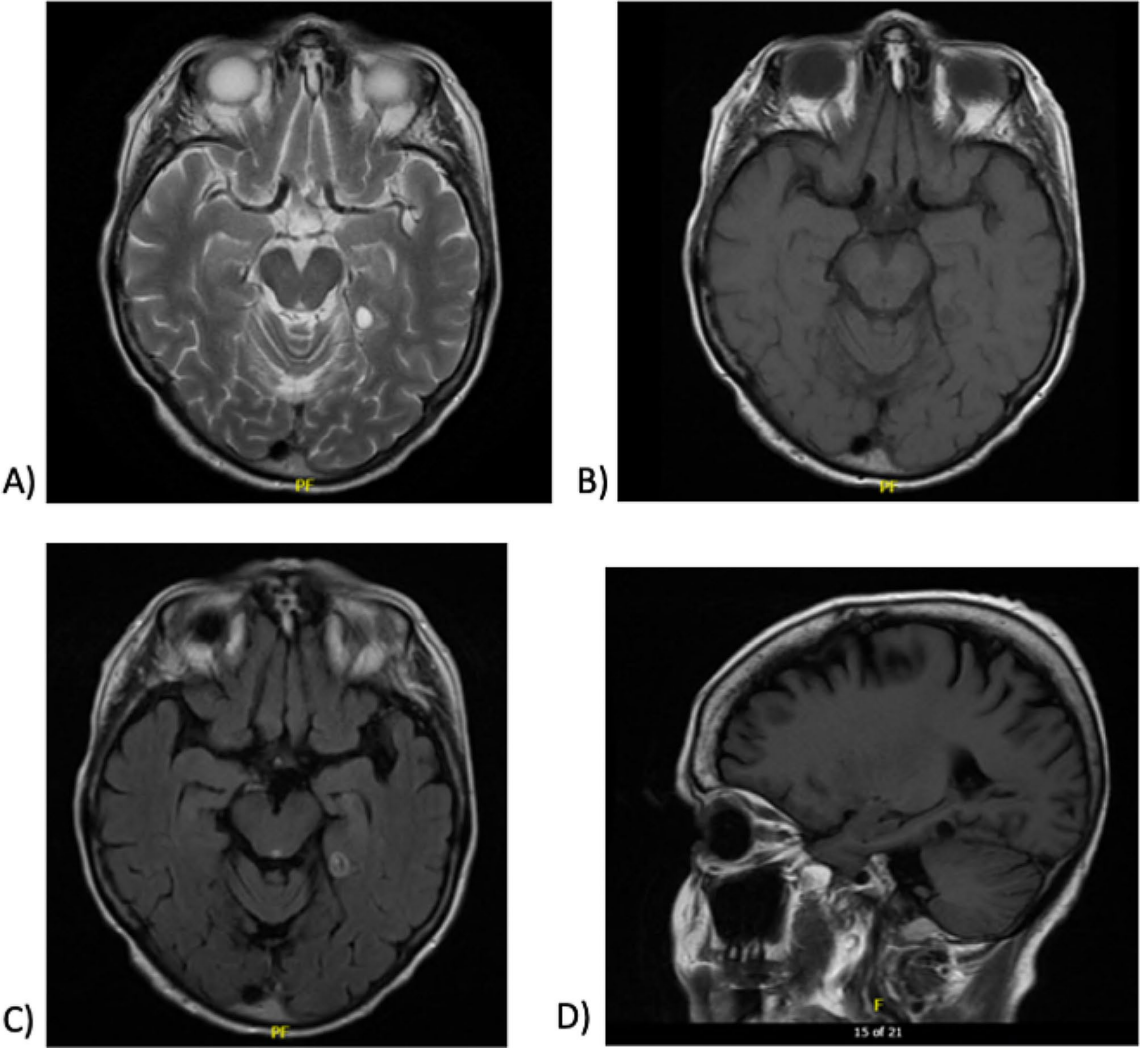
MRI Brain, November 2022, demonstrates the left ring enhancing posterior temporal lesion in the parahippocampal gyrus to medial occipitotemporal gyrus region, visible on imaging since September 2022. (**A**) Axial T2 Propellor. (**B**) Axial T1 Fast Spin Echo. (**C**) Axial T2 FLAIR post-gadolinium. (**D**) Sagittal T1 FLAIR

As of September of 2022, the patient had been on her combination of systemic maintenance treatments for approximately 6 months; repeat imaging for re-staging showed a new 6 mm lesion in the left posterior temporal lobe which was seen to increase in size by late November of 2022 (Fig. 2). Around this time, she was admitted to hospital for symptomatic management, and ultimately decided to discontinue systemic therapy targeting her cancer.

According to the patient, this is around the time when she first noticed her dissociative symptoms. When asked to retrospectively rate her symptoms as they felt at this time, she scored a 5 on the Dissociative Experiences Scale– II (DES-II) [23, 24], with the majority of her symptoms reflecting depersonalization and derealization (score of 3.6 on depersonalization and derealization factor items). She was started on 4 mg daily oral dexamethasone in mid-November and underwent stereotactic radiation to the left temporal brain metastasis in mid-December. Her dexamethasone was then increased to 12 mg in mid-December, before being tapered back to 4 mg over the following four weeks. In mid-January of 2023, the dexamethasone was increased back to 8 mg, as the patient was describing distressing symptoms of depersonalization and derealization. For example, *“a sensation of not being connected to her body… new over the past few months and has been occurring intermittently, but more pervasive in the past few days”* as well as describing a *“feeling of being dissociated and a sensation that her voice is not her voice, and that she feels separate from her body. She does not feel particularly anxious and denies the sensation of being tied into any feelings of anxiety or stress.”* These experiences did not represent true out-of-body experiences, rather depersonalization episodes wherein the patient felt estranged from her own body and experiences. Similarly, the patient described episodes of derealization, where she felt detached from her external reality.

At a follow-up visit several days later, it was noted that these symptoms had improved with the increased dose of dexamethasone. By the time of assessment by writers in early February, her DES-II score had decreased to 0.71 (score of 0 on depersonalization and derealization factor items); at this assessment she was denying any symptoms of dissociation, or any other associated symptoms. She stated that when she had been experiencing the depersonalization and derealization previously, the episodes had lasted for approximately one to two hours at a time and were intermittent throughout the day, but did occur on most days. Throughout, her presentation was inconsistent with previously documented cases of autoimmune encephalitis associated with immunotherapy [25], and there was no evidence of stroke on neuroimaging.

The patient remained well with respect to her symptoms of dissociation for several weeks. Her dexamethasone was decreased to 6 mg daily in the beginning of March, and subsequently decreased again to 4 mg daily by the end of the month. Her dissociative symptoms then returned, for example as she *“continues to have a sense of being outside of her body and difficulty focusing.”* She was offered a trial of methylphenidate 5 mg in the morning to try to assist with this symptom as well as her energy and lack of focus. Sadly, within the next two weeks her overall cancer symptom burden (especially pain) had worsened to the point where she required an admission to the palliative medicine service (unrelated to the methylphenidate), and she chose to undergo medical assistance in dying shortly thereafter.

### Background medical, surgical, psychiatric, and social history

Other than her cancer, the patient’s past medical history was notable for immune-mediated arthritis while on pembrolizumab as of early 2022; diverticulitis; osteoarthritis; remote history of ‘non-A, non-B hepatitis’ leading to hospital admission over 25 years ago; myopathy of unknown origin following bronchoscopy with general anesthesia approximately ten years ago, which resolved with steroids over the course of a few days; COVID-19 in early 2022; and varicose veins. She had no history of head injury, seizure, or stroke. The patient had finished preliminary college and was previously employed full-time but had since retired. She had a 29 pack-year history of smoking, having quit at the time of her cancer diagnosis, and did not regularly use any other recreational substances other than the occasional glass of wine.

As a part of her assessment, the patient underwent a semi-structured interview using the Structured Clinical Interview for the Diagnostic and Statistical Manual of Mental Disorders, 5th edition (DSM-5; SCID-5). She denied any current or previous history of symptoms consistent with any psychiatric disorder other than the depersonalization and derealization detailed in this report. Her mental status exam at time of assessment was grossly normal. She had no previous psychiatric diagnoses and no family history of any psychiatric conditions. She was right-handed. Complete neurological examination was grossly normal, other than that her voice was somewhat hoarse, and she endorsed some subjective memory complaints, though nothing objectively notable or that impaired the course of the assessment. Her motor exam was only notable for limited bilateral hip flexion due to pain (4/5 bilaterally). She did have a slight intention tremor in her left hand, which she indicated was not new.

## Discussion and conclusions

We presented here the case of a 68-year-old woman with recurrent non-small cell lung cancer, with ring-enhancing metastatic lesion to her left posterior temporal lobe leading to symptoms of depersonalization and derealization in the absence of any other psychiatric pathology. Her reasonably large (6 mm +) left-sided posterior temporal lobe lesion in the parahippocampal gyrus to occipitotemporal gyrus region appearing on MRI corresponded temporally to the commencement of her clinical symptoms of depersonalization and derealization, which then resolved with an increase in her dexamethasone, presumably as peri-tumoral edema decreased. Symptoms returned as her disease course worsened (we posit– though there was no further imaging, given her palliative course), and as dexamethasone dose was decreased.

Neurobiologically, depersonalization and derealization are hypothesized to arise from a vestigial “hard wired” distributed network, evolutionarily developed to increase attention and decrease emotional response in life-threatening situations, in order to enhance performance and theoretically increase survival especially in situations where the perceived threat cannot be localized [8]. This is thought to occur via simultaneous activation of several parallel processes, connecting medial prefrontal cortices (mPFC), as well as somatosensory and association cortices, with the limbic system via the amygdala [8, 26]. Firstly, features of decreased emotional response to stimuli arise when signals from the (primarily left) mPFC (and likely also primary somatosensory and related association cortices) inhibit the amygdala, which then in turn has inhibitory effects on the anterior cingulate cortex (ACC), leading to decreased sympathetic response [8, 26]. Secondly, as the left mPFC inhibits the amygdala, there is decreased autoinhibition within the amygdala, leading to excitatory output to ascending arousal systems. These, in addition to creating a state of increased vigilant attention, also increase inhibitory output to the ACC from the right mPFC, further decreasing emotionality. In combination, this state of increased vigilant attention with simultaneously decreased emotionality (i.e. increased stimulus detection but decreased related affective response) is posited to phenomenologically present as symptoms of depersonalization and derealization (as well as associated symptoms, such as reduced autonomic output) [8, 26].

Activation of these circuits represents a type of functional sensory-limbic disconnection [26], where emotional response is unlinked from sensory input, which then precipitates symptoms of depersonalization and derealization. This functional disconnection is thought to be what occurs in ‘psychiatric’ depersonalization; in trauma- and stressor-related disorders, or panic disorders, for example, it is thought that once a threshold level of anxiety is hit (hijacking the “non-localizable threat” pathways as described above), the left medial prefrontal cortex inhibits amygdala function via the above described mechanisms, beginning the pathway [8, 26]. However, physiologic limbic disconnection syndromes (and seizures) can also lead to experiences similar to depersonalization, via disruption in pathways connecting emotional experiences to input stimuli. For example, right or bilateral occipitotemporal lesions can impair patients’ ability to connect emotional responses to visual stimuli, likely via disruption of pathways connecting primary visual from limbic structures (8, 26).

**Fig. 3:**
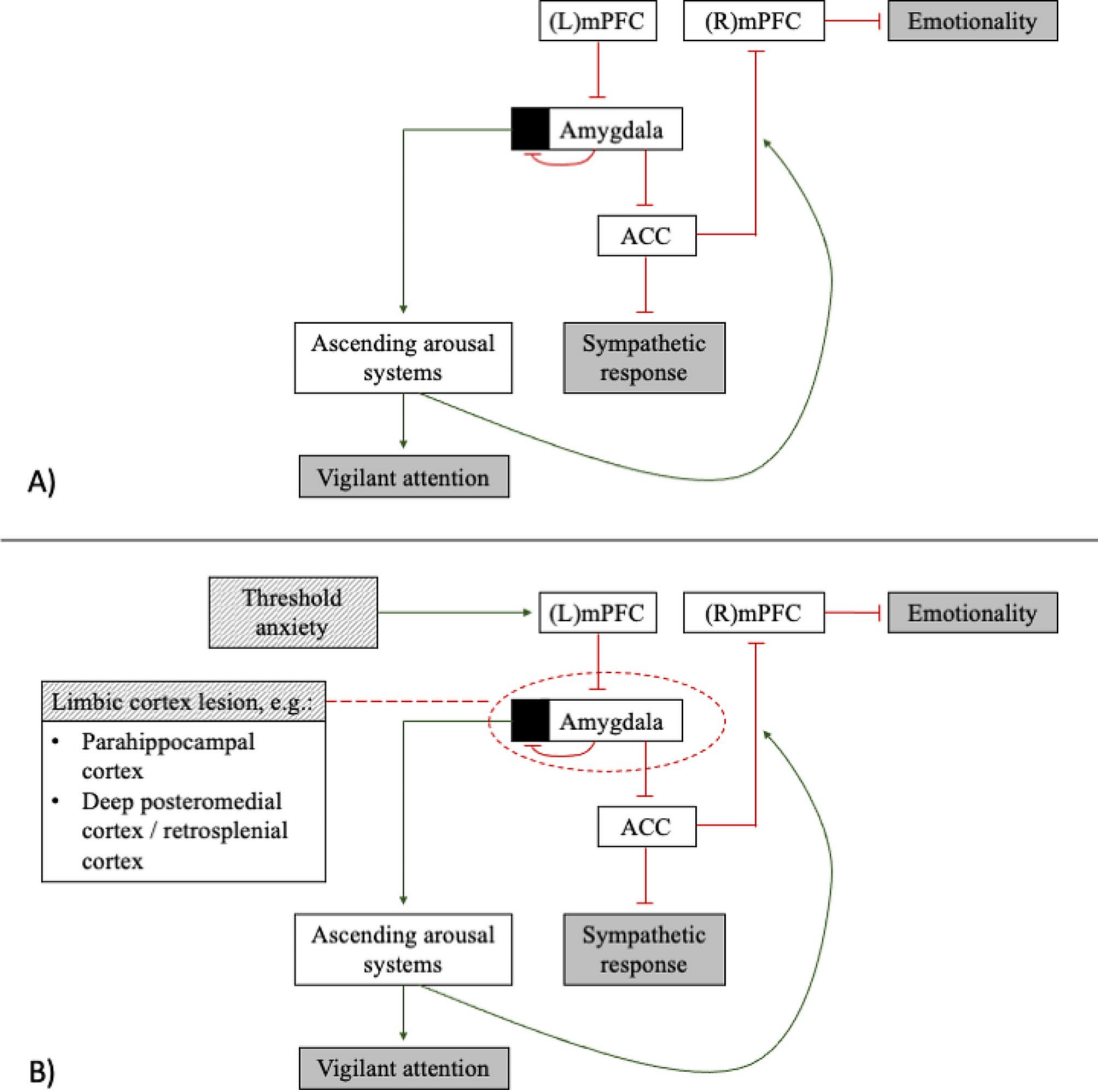
Diagram of potential network of depersonalization and derealization (as a result of decreased emotionality and sympathetic response, with increased vigilant attention), adapted from [8]. (**A**) Proposed network of depersonalization and derealization. (**B**) Proposed network with indications of where primary psychiatric causes (i.e. threshold anxiety) and limbic cortex lesions (as in our case) may influence the network, leading to symptomatology. Red lines indicate decreased output, green arrows indicate increased output

In the present case, we see symptoms of depersonalization and derealization present with the occurrence of a lesion in the left posterior limbic lobe, in the region of the parahippocampal gyrus to medial occipitotemporal gyrus. In our patient, it is most likely that the limbic cortex lesion structurally disrupted the pathways outlined above, and interfered with the structures therein, resulting in her symptoms. This points to the importance of the parahippocampal and occipitotemporal gyri in these phenomena. Indeed, prior research has demonstrated that parahippocampal cortex is important for contextual grouping of visual stimuli, as part of the cortical contextual network, as well as associative processing more generally [27]. The parahippocampal cortex has also been shown to potentially signal violations of typical associations [27]. The parahippocampal cortex receives projections from multiple areas, including retrosplenial cortex, and is important for spatial memory and retrieval of associations (as is potentially the retrosplenial cortex itself, which is also connected to the amygdala, though this area may represent familiar associations at a different level of abstraction than the parahippocampal gyrus) [27–29]. Rhythmic activation of the retrosplenial cortex, and coupling of same with related structures in the thalamus (with simultaneous uncoupling from other cortical regions, such as the somatosensory cortex and subiculum) has also been implicated in the pathogenesis of dissociative experiences in mice models [30]. Activation of deep posteromedial cortex (a core element of the default mode network, comprised of retrosplenial, posterior cingulate, and medio-ventral precuneus cortices, which has strong connections to ACC), especially on the left, has been similarly liked to human cases of dissociative experiences [29, 30] (Fig. 3).

In our patient, the metastatic lesion in question likely disrupted connections between occipitotemporal, retrosplenial, and parahippocampal regions with associated structures such as amygdala and cingulate cortex, in addition to decreasing functionality of the tumor region itself, leading to experiences of depersonalization and derealization. This case serves as a unique contribution to the literature on the neurobiology of these phenomena, given the specific localization of the intracerebral pathology and temporal specificity of symptoms relative to tumor growth and treatment course. However, there are limitations to this case report. We were not able to complete any neurocognitive testing with the patient, which may have been informative, especially given the location of the lesion. Additionally, as the patient did not undergo EEG (given the palliative stage of her illness), we were unable to definitively rule out seizure activity. However, clinically, she did not present with any other symptoms suggestive of seizure, such as aura, déjà vu, or any generalizing symptoms, and denied any symptoms consistent with a post-ictal state. Additionally, we feel that even if there was some limited local seizure activity secondary to her lesion, this case is still valuable from a localization standpoint, as pointing to the likely functional neurobiology involved in depersonalization and derealization.

Overall, the case of the patient discussed herein is unique, as it is one of the only to the authors’ knowledge describing isolated depersonalization and derealization in the absence of clinical seizure activity or other psychiatric symptomatology, attributable to a specific intracerebral lesion in the parahippocampal to occipitotemporal gyrus region.

## Data Availability

Data sharing is not applicable to this article as no datasets were generated or analysed during the current study.

## Footnotes

1 The patient had a non-small cell lung cancer (moderately-poorly differentiated adenocarcinoma).

## Abbreviations

ACC: Anterior Cingulate Cortex
DES-II: Dissociative Experiences Scale– II
DSM-5: Diagnostic and Statistical Manual of Mental Disorders, 5th edition
EEG: Encephalography
mPFC: Medial Prefrontal Cortex
SCID-5: Structured Clinical Interview for the DSM-5
